# The Multi-allelic Genetic Architecture of a Variance-Heterogeneity Locus for Molybdenum Concentration in Leaves Acts as a Source of Unexplained Additive Genetic Variance

**DOI:** 10.1371/journal.pgen.1005648

**Published:** 2015-11-23

**Authors:** Simon K. G. Forsberg, Matthew E. Andreatta, Xin-Yuan Huang, John Danku, David E. Salt, Örjan Carlborg

**Affiliations:** 1 Department of Clinical Sciences, Division of Computational Genetics, Swedish University of Agricultural Sciences, Uppsala, Sweden; 2 Institute of Biological and Environmental Sciences, University of Aberdeen, Aberdeen, Scotland, United Kingdom; The University of North Carolina at Chapel Hill, UNITED STATES

## Abstract

Genome-wide association (GWA) analyses have generally been used to detect individual loci contributing to the phenotypic diversity in a population by the effects of these loci on the trait mean. More rarely, loci have also been detected based on variance differences between genotypes. Several hypotheses have been proposed to explain the possible genetic mechanisms leading to such variance signals. However, little is known about what causes these signals, or whether this genetic variance-heterogeneity reflects mechanisms of importance in natural populations. Previously, we identified a variance-heterogeneity GWA (vGWA) signal for leaf molybdenum concentrations in *Arabidopsis thaliana*. Here, fine-mapping of this association reveals that the vGWA emerges from the effects of three independent genetic polymorphisms that all are in strong LD with the markers displaying the genetic variance-heterogeneity. By revealing the genetic architecture underlying this vGWA signal, we uncovered the molecular source of a significant amount of hidden additive genetic variation or “missing heritability”. Two of the three polymorphisms underlying the genetic variance-heterogeneity are promoter variants for Molybdate transporter 1 (*MOT1*), and the third a variant located ~25 kb downstream of this gene. A fourth independent association was also detected ~600 kb upstream of *MOT1*. Use of a T-DNA knockout allele highlights *Copper Transporter 6*; *COPT6* (*AT2G26975*) as a strong candidate gene for this association. Our results show that an extended LD across a complex locus including multiple functional alleles can lead to a variance-heterogeneity between genotypes in natural populations. Further, they provide novel insights into the genetic regulation of ion homeostasis in *A*. *thaliana*, and empirically confirm that variance-heterogeneity based GWA methods are a valuable tool to detect novel associations of biological importance in natural populations.

## Author Summary

Most biological traits vary in natural populations, and understanding the genetic basis of this variation remains an important challenge. Genome-wide association (GWA) studies have emerged as a powerful tool to address this challenge by dissecting the genetic architecture of trait variation into the contribution of individual genes. This contribution has traditionally been measured as the difference in the phenotypic means between groups of individuals with alternative genotypes at one, or multiple loci. However, instead of altering the trait mean, certain loci alter the variability of the trait. Here, we describe the genetic dissection of one such variance-controlling locus that drives variation in leaf molybdenum concentrations amongst natural accessions of *Arabidopsis thaliana*. The variance-controlling locus was found to result from the contributions of multiple alleles at multiple loci that are closely linked on the chromosome and is a major contributor to the “missing heritability” for this trait identified in previous studies. This illustrates that multi-allelic genetic architectures can hide large amounts of additive genetic variation, and that it is possible to uncover this hidden variation using the appropriate experimental designs and statistical methods described here.

## Introduction

Genome Wide Association (GWA) analysis is a powerful approach to study the genetic basis of complex traits in natural populations. It is widely used to study the genetics of human disease, but is equally useful in studies of other populations. For example, it has been used to dissect the genetics of traits of importance in agricultural applications (see e.g. [1] for an example in cattle) and ecological adaptation using collections of natural accessions in the genetic model plant *Arabidopsis thaliana*, for example [2–7].

The standard GWA approach screens the genome for loci where the alternative genotypes differ significantly in the mean for the trait or traits of interest. Although hundreds of loci have been found to affect a variety of quantitative traits using this strategy, it has become clear that for most complex traits this additive approach fails to uncover much of the genetics contributing to the phenotypic variation in the populations under study. It is therefore important to explore the genetics of such traits beyond additivity [8]. An alternative way that genetic variation can contribute to the phenotypic variability in a population is via direct genetic control of the variance [9]. To identify an individual locus that makes such direct contributions to the trait variance, a statistical test is used to identify significant differences in the phenotypic variance between the groups of individuals that carry alternative alleles at the locus. When such a variance difference exists between the genotypes at a locus, the locus displays a genetic variance-heterogeneity. These loci are therefore often referred to as variance-heterogeneity loci (or vQTL for short [10]). By performing genome-wide analyses to identify such variance-heterogeneity loci, novel trait associations and alternative genetic mechanisms involved in shaping the total phenotypic variance in the analyzed populations can be identified [8,10].

The direct genetic control of the phenotypic variance is a topic that has been studied for many years in quantitative genetics with a primary focus on its potential contributions to adaptation in natural populations and agricultural selection programs. Theoretical and empirical work has increased our understanding of how individual loci that display variance, rather than mean, differences between genotypes might cause phenomena such as fluctuating asymmetry, canalization and genetic robustness [9,11]. Empirical work now also supports the general principle that a direct genetic control of the variance is an inherent feature of biological networks and individual genes (see [12] for a review) and that it contributes to both capacitation [13,14] and maintenance of developmental homeostasis [15]. Although it was already shown in the 1980s that it was possible to map vQTL [16], this approach has only recently been more widely adopted to explore the role of variance-heterogeneity loci in, for example, environmental plasticity [15], canalization [17], developmental stability [18], and natural variation in stochastic noise [19].

With the advent of GWA analysis, and the later realization that standard additive models leave much of the genetic variance in the analyzed populations uncovered [8], there has been an increased interest in exploring the contribution of genetic variance-heterogeneity to the phenotypic variability in complex traits [10,20]. Several recent studies in, for example, humans [21], plants [7,19,22], *Drosophilia melanogaster* [23] and yeast [24] have shown that part of this previously unexplored heritable genetic variation, beyond the narrow-sense heritability, can be uncovered by re-analyzing existing GWA datasets using methods to detect differences in trait variance (variance-heterogeneity GWA or vGWA for short) between genotypes [20–22].

Previously, we re-analyzed ionomic data from a GWA study based on 93 wild-collected *A*. *thaliana* accessions [2] and detected a variance-heterogeneity locus with a genome-wide significant difference for the variance in leaf molybdenum concentrations between the genotypes. This association was found near the *MOT1* (Molybdate transporter 1) gene [22]. Importantly, this locus did not affect the mean leaf molybdenum concentrations in this dataset [2,22]. Molybdenum is an essential element for plant growth due to its role as a part of the molybdopterin cofactor that is required by several critical enzymes [25]. Both deficiency and excess of molybdenum have an impact on plant development [26]. The ability of plants to acquire minerals from the soil, and regulate their levels in the plant, depends on complex biochemical and regulatory pathways. The genetic architecture of such ionomics traits is thus complex [27]. To date, several studies in *A*. *thaliana* have exploited natural variation and QTL analysis to examine mineral content [28–34], and important insights have been gained into the underlying biological mechanisms by dissecting the molecular determinants for nine of these QTL. These include QTL for the accumulation of Co, Mo, Na, Cd, As, S/Se, Zn, Cu and sulfate [5,6,35–42]. Further, GWA analysis has also been used to identify both candidate loci and functional polymorphisms contributing to natural variation in these ionomics traits [2,3,5,6,43].

Here, we quantified molybdenum concentrations in leaves in a larger collection of 340 natural *A*. *thaliana* accessions to replicate and dissect the genetic architecture of the previously detected variance-heterogeneity locus around the *MOT1* gene [22]. We uncovered that a complex multi-locus, multi-allelic genetic architecture leads to the genetic variance-heterogeneity at this locus. Several polymorphisms in three closely linked loci were significantly associated with the mean molybdenum concentration in the leaf, and due to an extended LD between the minor alleles at these loci, their joint effects cause the genetic variance-heterogeneity at this locus. By dissecting this variance-heterogeneity locus in detail, we both reveal the genetic complexity of an adaptive locus for molybdenum homeostasis in *A*. *thaliana* [37] and uncover a significant amount of novel additive genetic variance that otherwise would remain undetected and contribute to the “missing heritability”.

## Results

### An increased population-size reveals novel loci associated with molybdenum concentrations in *A*. *thaliana* leaves

**Fig 1:**
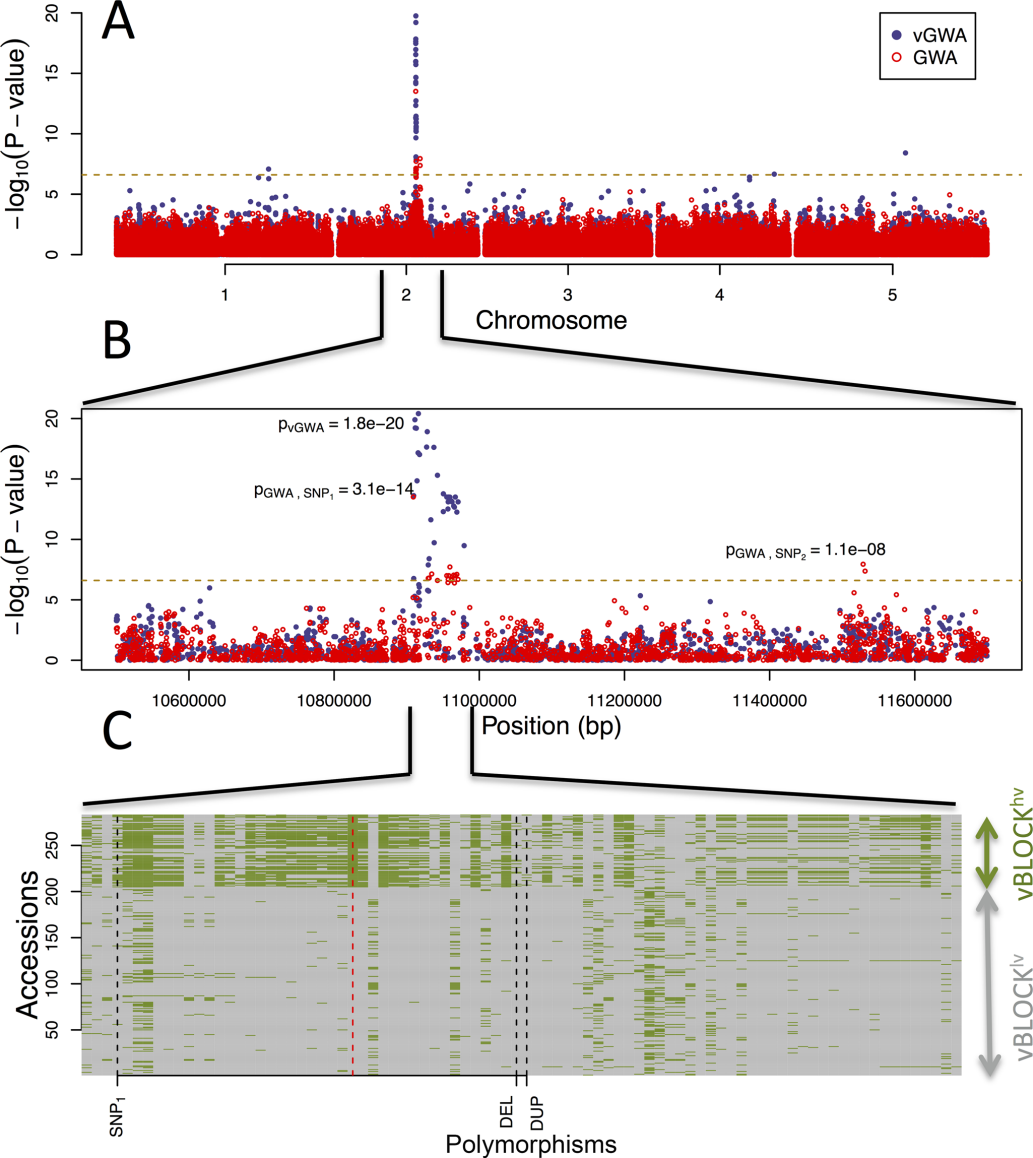
GWA and vGWA analyses for mean leaf molybdenum concentration. (A) Genome-wide results from single-locus vGWA (blue) and GWA (red) analyses across the *A*. *thaliana* genome. (B) Region on chromosome 2 where a highly significant genetic variance-heterogeneity was detected for the leaf molybdenum concentrations. Several significant SNPs are detected and these define an extended vGWA associated region (vBLOCK), where the minor alleles at these significant loci define an LD-block associated with a higher phenotypic variance (*vBLOCK^hv^*). (C) Illustration of the high LD across vBLOCK. The accessions that are homozygous for the minor/major allele are colored green/grey and then sorted according to the genotype of the SNP with the strongest genetic variance-heterogeneity (red dashed line, Table 1).

The first GWA analysis searching for genetic effects on mean leaf molybdenum concentrations [2] did not uncover any genome-wide significant associations for this trait. This was surprising as it was known from earlier QTL studies that a strong polymorphism affecting this trait was segregating in the analyzed population [36]. To investigate this further we measured the molybdenum concentration in leaves from at least six replicate plants of 340 natural *A*. *thaliana* accessions (S1 Table) that had earlier been genotyped using the 250k *A*. *thaliana* SNP-chip [3]. 58 of the accessions used in this study overlapped with those in the previous study [2,22]. In this larger dataset, we detected several SNPs associated with the mean leaf molybdenum concentrations in, or near, the *MOT1* locus (Fig 1). The minor alleles for some associated SNPs increased the mean phenotype, whereas others decreased it relative to the major allele (Table 1; Fig 1B). In our earlier study we identified a genome-wide significant genetic variance-heterogeneity for leaf molybdenum concentrations at this same locus containing *MOT1* [22]. Here, we therefore aim to functionally dissect this region further to obtain a deeper understanding of the genetic mechanisms controlling the range of leaf molybdenum concentrations observed in *A*. *thaliana* [36].

**Table 1:** Mean and variance effects for five loci in the *MOT1* region associated with either mean molybdenum concentration levels (GWA) or variance (vGWA).

Locus	SNP^1^	Alleles^2^	Location (bp)^3^	Effect (95% CI)^4^	Significance^5^
**Mean associations**					
DEL	n.a.	*abs*/*pres* (4%)	10,934,564–10,934,616	-2.6 (-3.2–-2.0)	4.2 × 10^−16^
DUP	n.a.	*abs*/*pres* (3%)	10,934,814–10,935,143	2.8 (2.0–3.6)	5.0 × 10^−11^
SNP_1_	rs347469902	A/T (14%)	10909091	0.8 (0.4–1.2)	4.6 × 10^−5^
SNP_2_	rs347287517	A/C (19%)	11528777	1.0 (0.7–1.3)	3.0 × 10^−10^
**Variance association**					
vBLOCK	rs346654259^6^	A/G (29%)	10917720	7.1 (5.4–9.4)	5.6 × 10^−12^

^1^Name of the most significant associated SNP markers at each locus in the GWA (Mean associations) or vGWA (Variance association) analyses

^2^Major allele/Minor allele (minor allele frequency). For the structural variants, abs and pres denotes absence and presence of the variant respectively

^3^Location in the TAIR10 assembly

^4^Estimates of mean effects (μg Mo /g dry weight) and 95% Confidence Intervals (CI) for the minor alleles from a four-locus fixed-effect regression model including DEL, DUP, SNP_1_ and SNP_2_, and the variance effect (fold increase in variance for the minor allele) for vBLOCK from a DGLM model

^5^Significances for the effects estimated in^4^

^6^Top SNP identified in DGLM scan of vBLOCK.

### Dissecting the genetic structure of a variance-heterogeneity locus affecting molybdenum concentrations in *A*. *thaliana* leaves

A vGWA analysis of leaf molybdenum concentrations in the 340 accessions, searching for genetic effects on the between accession variance heterogeneity (S1 Text), revealed several SNP markers that displayed a genome-wide significant genetic variance-hetereogeneity in the region of the reported vQTL near the *MOT1* gene [22]. The associations were particularly strong (Fig 1A) for a number of SNPs in high LD on chromosome 2 (Fig 1B; vBLOCK). By visualizing the genotypes for the analyzed accessions across vBLOCK, we observed that the population contains two distinct multi-locus genotype classes for this segment: one that predominantly contains high-variance associated SNP alleles (*vBLOCK* ^*hv*^) and another with low-variance associated SNP alleles (*vBLOCK* ^*lv*^; Fig 1C). vBLOCK contains in total 20 annotated genes, and the most obvious functional candidate for the association is *MOT1* (10,933,061–10,934,551).

### Multiple structural *MOT1* promoter-polymorphisms are associated with molybdenum concentrations in *A*. *thaliana* leaves

**Fig 2:**
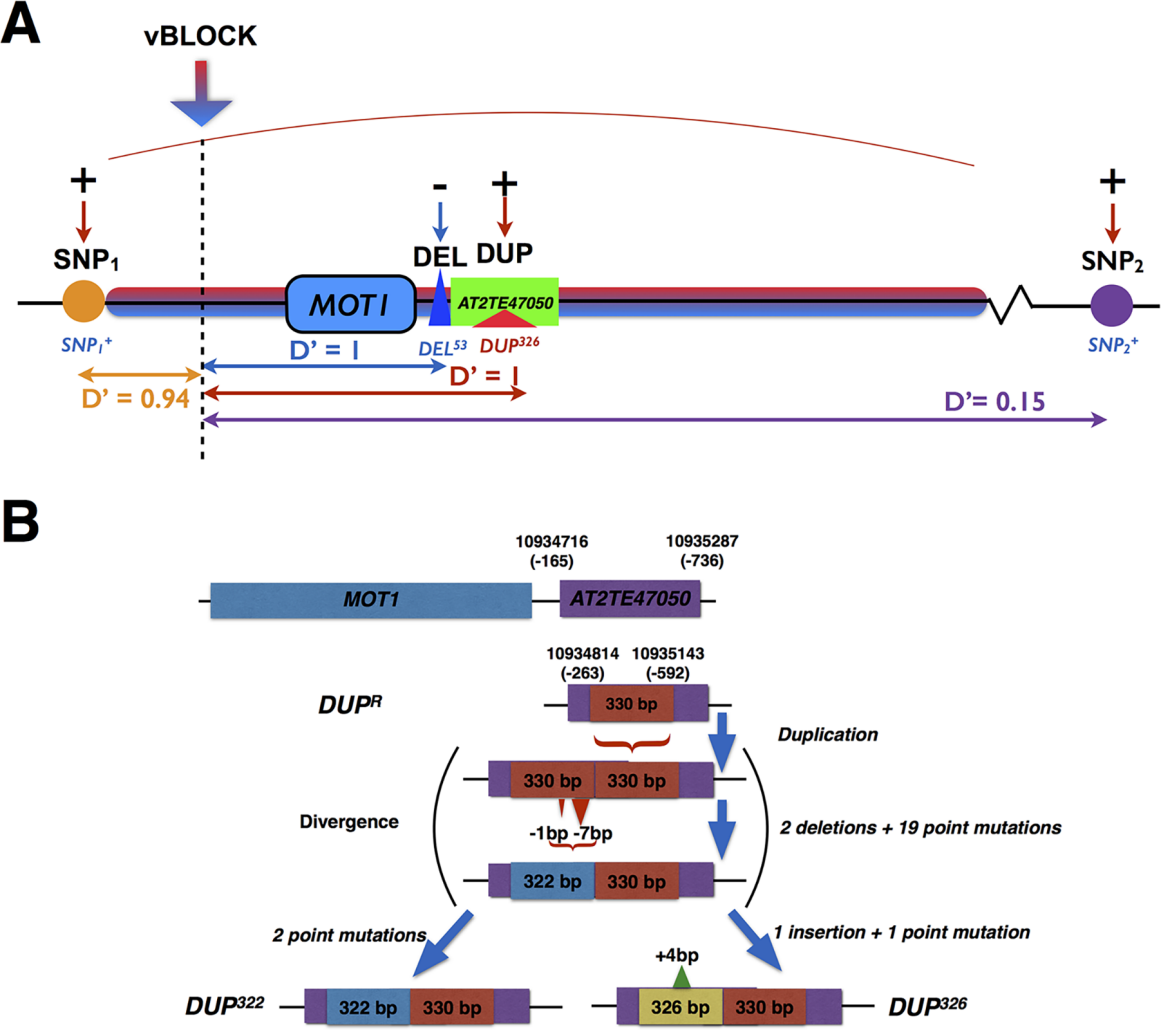
Schematic illustration of the complex locus on chromosome 2 associated with leaf molybdenum concentrations. **(A)** Multiple GWA and vGWA signals were detected to a complex locus around *MOT1*. There was a strong LD (D’) between three of the associated loci (SNP_1_, DEL and DUP) and the high-variance associated variant of vBLOCK (*vBLOCK* ^*hv*^) that led to the extended vGWA signal (Red/blue arrow indicate leading vGWA SNP in the DGLM analysis). A fourth independent GWA association (SNP_2_) was also detected upstream of vBLOCK. The direction of the effects for the minor alleles at the significantly associated loci (*SNP1* ^*+*^, *SNP2* ^*+*^, *DEL* ^*53*^ and *DUP* ^*326*^) relative to that of the major, reference allele are illustrated with + (increased) and—(decreased), respectively. In **(B)** we illustrate the differences between the reference allele at DUP (*DUP* ^*R*^) and the two variants of the 330 bp duplication (*DUP* ^*326*^ and *DUP* ^*322*^) in the transposable element *AT2TE47050* in the promoter region of *MOT1*.

*MOT1* is an obvious functional candidate gene for the genetic variance-heterogeneity for vBLOCK. A 53 bp deletion in the promoter-region of this gene has earlier been shown to decrease *MOT1* expression, leading to low concentrations of molybdenum in the plant [36,44]. To complement our SNP-marker dataset with this known, and other potentially functional, structural promoter polymorphisms segregating in the analyzed population, we screened the promoter region of *MOT1* using PCR fragment size differentiation (see Methods for details) and identified in total six non-coding structural polymorphisms (Fig 2, S1 Table). These were then genotyped in 283 of the 340 phenotyped accessions.

Two of the six segregating *MOT1* promoter polymorphisms were significantly associated with mean leaf molybdenum concentration. The first was *DEL* ^*53*^ which is located 13 bp upstream from the transcription start-site of *MOT1*. Baxter *et al*. [36] earlier showed that this 53 bp deletion (*DEL* ^*53*^) allele lacks the TATA-box in the *MOT1* promoter, which leads to a reduced expression of *MOT1* and decreased molybdenum concentration in the leaf. We confirm that this allele decreased the mean molybdenum concentrations in the leaf also in this dataset (Table 1; p_nominal_ = 4.2x10^-16^; Fig 2A) and found the *DEL* ^*53*^ allele only among low molybdenum accessions (Mo < 3 μg g^-1^ dry weight). We also found a strong association (p_nominal_ = 5.0x10^-11^; Table 1; Fig 2A) to a locus (DUP) located 263 bp upstream from the translation start site. Here, several accessions share a 330bp long duplication (Fig 2B) located inside a transposable element (*AT2TE47050*). The duplication exists in two distinct variants (alleles) differing by four polymorphisms: three point-mutations and one 4bp insertion (*DUP* ^*326*^ and *DUP* ^*322*^ in Fig 2B). In our dataset, the *DUP* ^*326*^ allele altered leaf molybdenum concentrations and it was found only among accessions with high leaf molybdenum concentrations (Mo > 10 μg g^-1^ dry weight). To our knowledge, this duplication has not previously been described in the literature. Using qRT-PCR, we tested the *MOT1* expression in 5 accessions carrying the low-molybdenum *DEL* ^*53*^ allele and found that 4 of these have significantly lower expression than Col-0 in the root (95% CI 0.2–0.6 fold; 2.5 × 10^−15^ < p < 2.5 × 10^−3^ from Fishers method combining p-values for the biological replicates; S3 Table). Using the same assay, we tested 6 accessions carrying the high-molybdenum *DUP* ^*326*^ allele. All these accessions had higher (95% CI 2.2–7.8 fold; 2.5 × 10^−23^ < p < 2.2 × 10^−3^ from Fishers method combining p-values for the biological replicates; S3 Table) *MOT1* expression than Col-0 in the root. Although these results do not provide direct functional evidence that the *DUP* ^*326*^ allele increases the molybdenum concentration in the leaves via an increased expression of *MOT1* in the roots, it suggests this as a plausible mechanism worth further explorations. Together, our results provide further evidence that allelic heterogeneity at *MOT1* is an important component of the genetic architecture of natural variation in leaf molybdenum concentrations.

### A multi-locus analysis confirms that a multi-locus, multi-allelic genetic architecture determines the molybdenum concentrations in plants from the global *A*. *thaliana* population

Multiple associations to loci with either mean- or variance differences between genotypes for leaf molybdenum concentrations were uncovered in the single-locus GWA and vGWA analyses. To confirm the independence of these effects, and evaluate their joint contributions to leaf molybdenum, we fitted all markers (SNPs and structural variants) on chromosome 2 in a generalized linear model to the mean leaf molybdenum concentration using the LASSO method [45]. This penalized maximum likelihood regresses the effects of polymorphisms that make no, or only a minor, independent contribution to the trait towards zero and highlights the markers that jointly make the largest contribution to the trait variation. The penalty in the analyses was chosen so that all highlighted polymorphisms in the final model also have a genome-wide significant effect in the earlier GWA or vGWA analyses (S1 Fig; see Methods section for details). In this way, the LASSO method picks up the genome-wide significant polymorphisms that have independent effects on the trait.

The *MOT1* promoter polymorphisms DEL and DUP were the most strongly associated loci in the LASSO analysis. Two additional SNP markers, one located ~25 kb downstream (rs347469902; 10,909,091 bp; SNP_1_; Table 1) and one ~600 kb upstream of *MOT1* (rs347287517; 11,528,777 bp; SNP_2_; Table 1), were also highlighted. The minor alleles at SNP_1_ and SNP_2_ (*SNP*_*1*_^*+*^ and *SNP*_*2*_^*+*^) were both enriched among accessions with high leaf molybdenum concentrations. The minor alleles at three of the four associated loci thus increased the mean leaf molybdenum concentrations (Table 1; *DUP* ^*326*^, *SNP*_*1*_^*+*^, and *SNP*_*2*_^*+*^), and one decreased it (Table 1; *DEL* ^*53*^).

### A multi-locus genetic architecture contributes to the range of molybdenum concentrations in wild collected *A*. *thaliana* accessions

Under certain conditions, multi-allelic genetic architectures can lead to a genetic variance-heterogeneity in association-analyses based on bi-allelic SNPs (see e.g. [10]). For example, if a locus contain a SNP with two alleles, *SNP* ^*A*^ and *SNP* ^*B*^, where the major SNP allele is completely linked to the major allele at gene *M* regulating trait *T* (i.e. only the *SNP* ^*A*^-*M* ^*WT*^ haplotype exists in the population). If now locus *M* also contains two minor alleles, *M* ^*-*^ and *M* ^*+*^, that decreases/increases *T* an equal amount relative to the value of *M* ^*WT*^, and that are tagged by the *SNP* ^*B*^ allele, the *SNP* ^*A*^ and *SNP* ^*B*^ genotype-classes will have identical means, but different variances. Here, we will show that the genetic variance-heterogeneity we detected for vBLOCK is due to a multi-allelic genetic architecture that closely resembles this example.

### An extended LD across three polymorphisms affecting mean molybdenum concentrations lead to a genetic variance-heterogeneity association in the vGWA analysis

There was a strong LD (D’) between three loci (SNP_1_, DEL and DUP) associated with the mean leaf molybdenum concentration and the SNPs across vBLOCK that displayed a highly significant genetic variance-heterogenity (Fig 2A; Table 2). All the 20 accessions carrying either the *DEL* ^*53*^ or *DUP* ^*326*^ alleles also carry the high-variance associated *vBLOCK* ^*hv*^. Of the 29 accessions that carry the high molybdenum *SNP*_*1*_^*+*^ allele, 19 carried *vBLOCK* ^*hv*^ (Fig 1C; see Methods section for further detail). The minor alleles at two of these (*DUP* ^*326*^, *SNP*_*1*_^*+*^) increased, and at one of them (*DEL* ^*53*^) decreased, the leaf molybdenum concentration. This results in a situation similar to that in the example above: multiple alleles with different directional phenotypic effects are unevenly distributed across the two variants of vBLOCK. The fact that one variant (*vBLOCK* ^*hv*^) tags three different minor alleles (*DUP* ^*326*^, *DEL* ^*53*^ and *SNP*_*1*_^*+*^) with different effects on the mean molybdenum concentration explains the increased phenotypic variance for this group of accessions.

**Table 2:** LD^c^ between the loci altering mean leaf molybdenum concentrations.

	DEL	DUP^a^	SNP_1_	SNP_2_	vBLOCK^b^
**DEL**	1	0	0.01	0	0.11
**DUP** ^**a**^	1	1	0.19	0.03	0.08
**SNP**_**1**_	1	1	1	0.03	0.34
**SNP**_**2**_	0.59	0.53	0.22	1	0.03
**vBLOCK** ^**b**^	1	1	0.94	0.15	1

^a^Minor allele in the analysis is *DUP* ^*326*^ that is associated with mean leaf molybdenum concentrations

^**b**^vBLOCK represented by the top-SNP in the dGLM scan across vBLOCK (Table 1)

^c^LD is provided as r^2^ /D’ above/below the diagonal, respectively.

**Fig 3:**
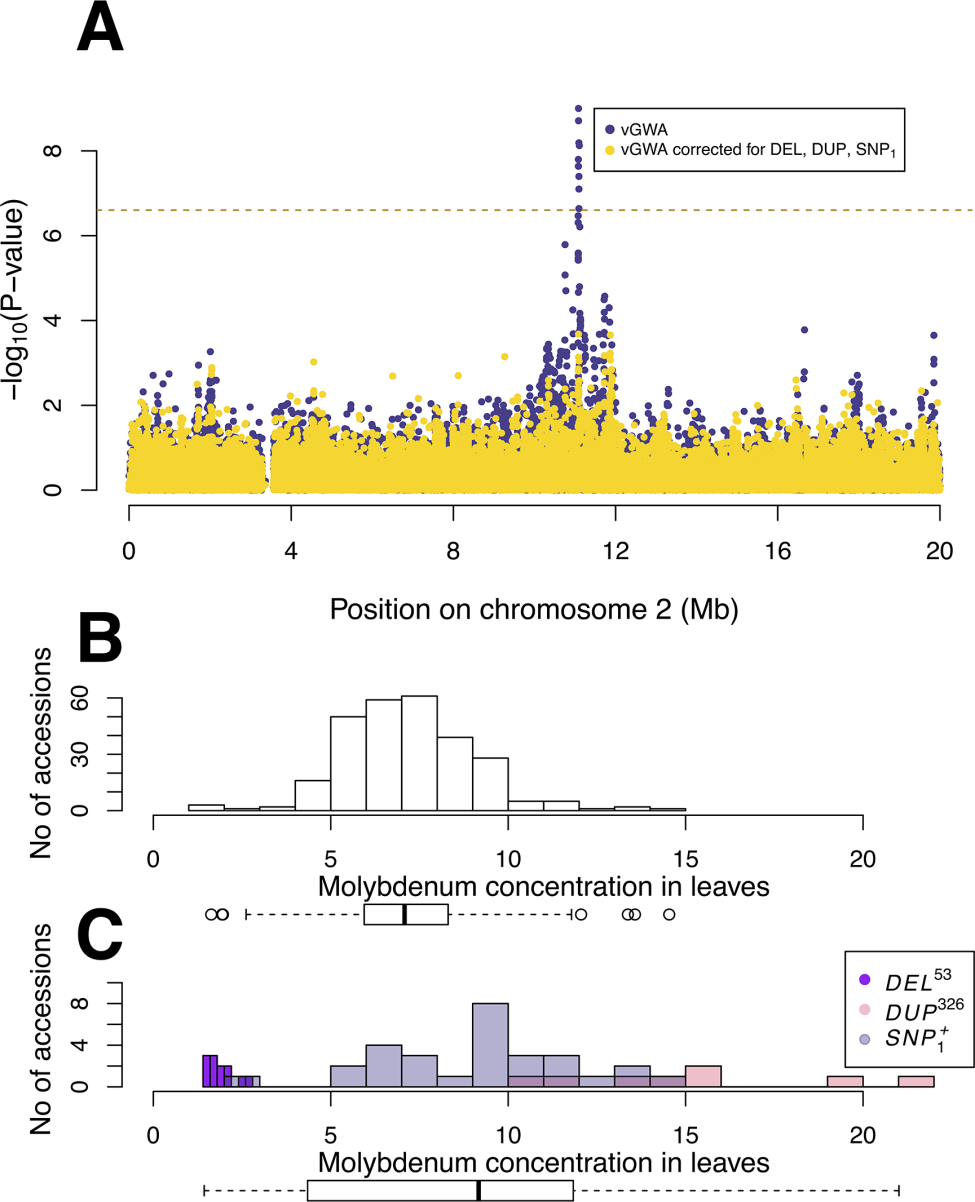
The genetic variance-heterogeneity across vBLOCK emerges from a multi-locus, multi-allelic genetic architecture. **(A)** The vGWA analysis using the alternative DGLM approach also detects a strong association near *MOT1* on chromosome 2 (blue dots). The genetic variance-heterogeneity at this locus is, however, cancelled when the mean effects of the *DEL* ^*53*^, *DUP* ^*326*^ and *SNP*_*1*_^*+*^ alleles are included in the DGLM model (yellow dots). The variance in the mean leaf molybdenum concentrations is lower for the group of accessions carrying the low-variance associated variant of vBLOCK (*vBLOCK* ^*lv*^) (**B**) than for the group of accessions carrying the high-variance associated variant (*vBLOCK* ^*hv*^) **(C)**. Separate colors are used for the accessions carrying the *DEL* ^*53*^ (purple), *DUP* ^*326*^ (red) and *SNP*_*1*_^*+*^ (grey) alleles in **(C)** to illustrate how these alleles generate the high variance in mean leaf molybdenum concentrations associated with *vBLOCK* ^*hv*^.

To statistically disentangle the genetic effects on the mean and variance by this multi-allelic, multi-locus genetic architecture, an additional vGWA analysis was performed where we fitted a linear model with separate effects for the mean and variance to the data as outlined by Valdar and Rönnegård [10]. The three mean associated loci that were located within vBLOCK (DUP, DEL and SNP_1_) were fitted as loci with mean effects when screening chromosome 2 for loci with potential effects on the variance using this method. The entire variance signal to vBLOCK disappears in this analysis (Fig 3A) illustrating that the variance-heterogeneity association to vBLOCK is due to the presence of the *DEL* ^*53*^, *DUP* ^*326*^ and *SNP*_*1*_^*+*^ alleles on the high-variance associated *vBLOCK* ^*hv*^ (Fig 3C).

### New additive genetic variation revealed by the dissection of a locus detected via its genetic variance-heterogeneity

We estimated the broad-sense heritability of leaf molybdenum concentrations from the within/between accession variances to be H^2^ = 0.80 using an ANOVA across all replicated measurements. This estimate is similar to that reported in earlier studies (0.56 [43] to 0.89 [2]). The narrow-sense heritability was estimated to be h^2^ > = 0.63 using a mixed model based analysis where the accession mean phenotypes were regressed onto the genomic kinship matrix.

The first GWA analysis for leaf molybdenum concentrations by Atwell *et al*. [2] was unable to detect any loci contributing to the variation in the trait mean. The later vGWA study by Shen *et al*. [22] identified a genetic variance-heterogeneity in the *MOT1* region that explained 27% of the phenotypic variance where the contribution by mean (additive) and variance (non-additive) effects were 4/23% of the phenotypic variance, respectively. Using the variance decomposition proposed by Shen *et al*. [22], we estimate that the genetic variance-heterogeneity at vBLOCK contributes 3 and 19% to the phenotypic variance via its effect on the mean and the variance. The total amount of genetic variance associated with the vGWA signal here is thus comparable to that of Shen *et al*. [22], but in both studies it leaves much of the total additive genetic variance unexplained as it only accounts for about 5% of h^2^. The contribution to H^2^ is, however, larger and between 24 to 28% in these two studies.

However, after considering the individual contributions made by the three polymorphisms identified on *vBLOCK* ^*hv*^ (*DEL* ^*53*^, *DUP* ^*326*^, *SNP*_*1*_^*+*^; Fig 3), much additive genetic variance is uncovered. Nearly all the contribution from *vBLOCK* becomes additive (83% of the total variance) to explain 45% of h^2^ and 43% of H^2^. By also accounting for the fourth locus (*SNP*_*2*_; Fig 2), the contribution h^2^ and H^2^ increases further to 60 and 50%, respectively. By dissecting the genetic architecture of the vGWA signal into its underlying multi-locus, multi-allelic components, we were thus able to reveal a significant contribution by vBLOCK to the “missing heritability” of molybdenum concentration in the leaf in the original GWA [2] and vGWA [22] analyses.

### Functional analyses of genes in LD with the loci affecting the mean molybdenum concentration in leaves

Here, we functionally explore the associations outside of the coding and regulatory regions of *MOT1* in more detail to identify additional functional candidate polymorphisms and genes for the regulation of molybdenum homeostasis.

### Mutational analyses to identify functional candidates contributing to variable leaf molybdenum concentrations in *A*. *thaliana*

Two regions outside of the coding and regulatory region of *MOT1* (chromosome 2 10,933,061–10,935,200 bp) were associated with the mean leaf molybdenum concentrations (*SNP*_*1*_ and *SNP*_*2*_ in Figs 1B; 3A). Genes located in the chromosomal regions covered by SNPs in LD (r^2^ > 0.4) with *SNP*_*1*_ and *SNP*_*2*_, respectively, were explored as potential functional candidates for the associations using T-DNA insertion alleles (S4 Table).

Four T-DNA alleles of five different genes in the region around *SNP*_*1*_ (10,909,091 bp; S2 Fig; S4 Table) were evaluated for leaf molybdenum concentrations, but in none of these did the leaf molybdenum concentrations differ from that of the wild-type Col-0.

**Fig 4:**
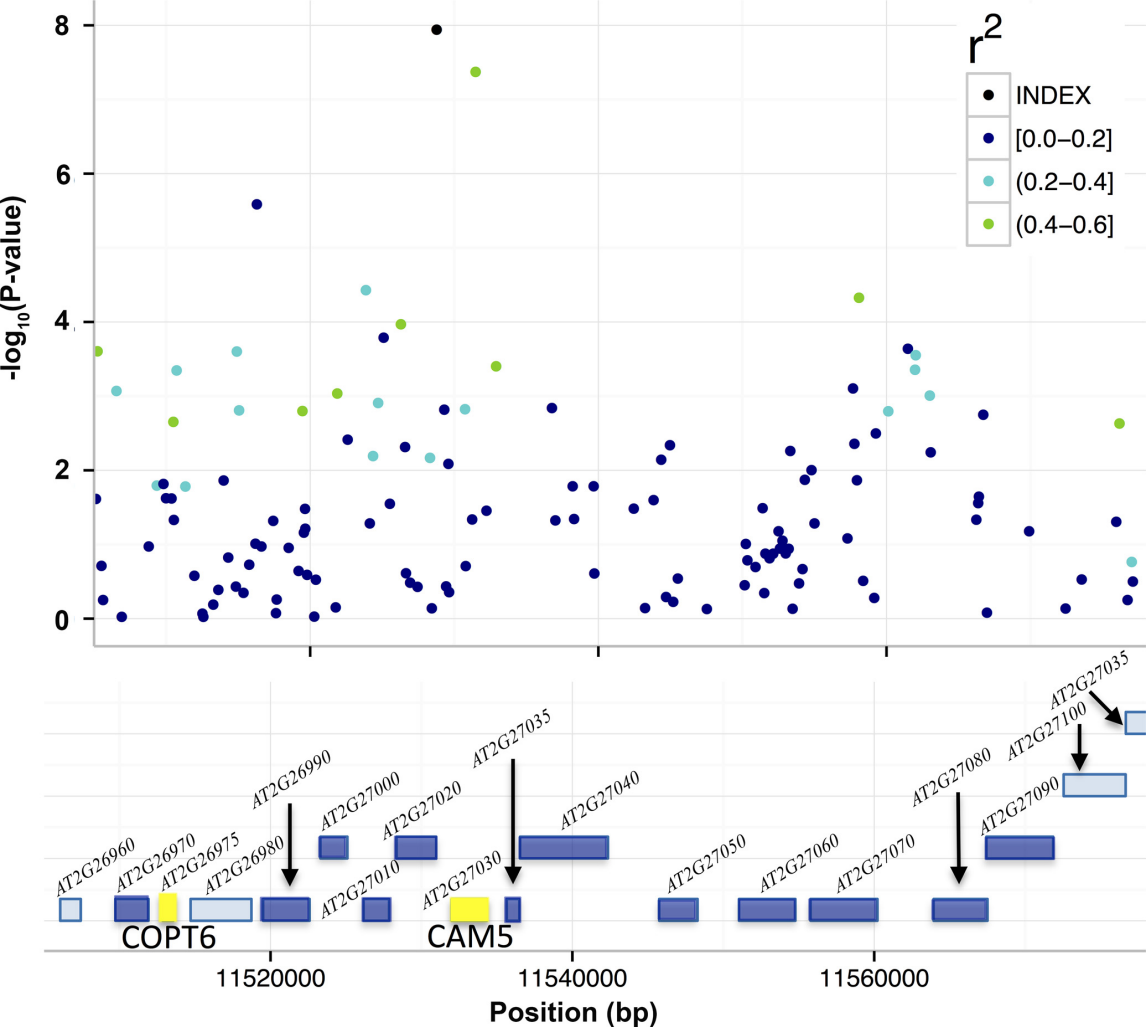
T-DNA analyses to identify candidate genes for the associations to mean leaf molybdenum concentrations. Included in the figure are the genes (colored boxes) in the region surrounding *SNP*_*2*_ that were bounded by the furthest up- and downstream SNPs with r^2^ > 0.4. We measured the mean leaf molybdenum concentrations for available T-DNA insertional alleles and compared them to the wild-type Col-0. Yellow box = significant difference in leaf molybdenum concentration, deep blue box = no significant difference, light blue = no T-DNA insertion line tested. The T-DNA lines with insertions in *AT2G26975* and between *AT2G27020/AT2G27030* had altered mean leaf molybdenum concentrations.

We also evaluated 19 mutants with T-DNA insertions in 14 genes around *SNP*_*2*_ (11,528,777 bp; Fig 4; S4 Table), and identified two with significantly altered leaf molybdenum concentrations compared to the wild-type Col-0 (Table 3). One (SALK_138758) has an insertion covering genes *AT2G27020* and *AT2G27030*, and the other (GK-350E02) has an insertion in gene *AT2G26975*. These T-DNA alleles showed on average 55 and 58% reductions in leaf molybdenum concentrations compared to wild-type Col-0, respectively (Table 3). *AT2G27020* was also evaluated via another T-DNA insertional allele (SAIL_760_D06), and this line had wild-type leaf molybdenum concentrations. Thus, *AT2G27030* (*ACAM2*/*CAM5*; 11,532,004–11,534,333) appears to be the most likely functional candidate gene of the two. Calmodulin is a known metalloprotein and a Ca^2+^ sensor, but no previous connections to molybdenum has been reported. The reduced leaf molybdenum concentration of the T-DNA insertional allele of *AT2G26975* (Copper Transporter 6; *COPT6*) makes this a second functional candidate locus for the association around *SNP*_*2*_. Interestingly, as well as low molybdenum, the T-DNA knockout allele of this gene has a slightly increased leaf copper concentration compared to wild-type (3.82 and 3.36 μg / g dry weight, respectively, in GK-350E02 and wild-type Col-0; p = 0.0018), suggesting a role of *COPT6* also in the regulation of copper homeostasis. From the literature it is known that copper and molybdenum homeostasis are related and that copper depleted *Brassica napus* plants have up-regulated expression of both copper transporter genes and *MOT1* [46].

**Table 3:** T-DNA insertion lines with significant associations to the mean leaf molybdenum concentrations.

T-DNA Line	ATG Number	Gene	Molybdenum concentration relative to Col-0^a^	n^b^	p-value^c^
SALK_138758	*AT2G27020;AT2G27030*	*PAG1*;*CAM5*	0.45	13 (24)^**d**^	0.008
GK-350E02^**e**^	*AT2G26975*	*COPT6*	Family 1: 0.50	17 (24)^**f**^	0.003
			Family 2: 0.33	12 (24) ^**f**^	0.001

^a^The ratio of leaf molybdenum concentration for the tested T-DNA insertion line relative to that of Col-0. Molybdenum concentrations are normalized against the wild-type Col-0 means in each growth tray

^b^Total number of biological replicates for the T-DNA insertional allele in the assay and in parentheses number wild-type Col-0

^c^Significance as determined by Wilcox rank test comparing molybdenum concentration in the leaves of the T-DNA insertional allele to that of wild-type Col-0

^d^Two independent experiments with 8/12 and 5/12 biological replicates of T-DNA allele/wild-type Col-0

^e^Tests made in two families grown from the selfed segregating parental line

^f^Two independent experiments with 7/12 and 5/12 biological replicates of the T-DNA allele/ wild-type Col-0

## Discussion

Common approaches to dissect the genetics of complex traits in segregating populations are linkage mapping and association studies. These studies aim to identify the loci in the genome where genetic polymorphisms control the phenotypic variance in the studied populations. This is achieved by screening for significant genotype-phenotype associations across a large number of genotyped polymorphic markers in the genome. The most common statistical models used in such analyses aim to identify loci with significant mean phenotype differences between the genotypes at individual loci. Although such models are powerful for capturing much genetic variance in populations, they have limited power when challenged with more complex genetic architectures including multiple-alleles, variance-heterogeneity and genetic interactions [8,47]. It is therefore important to also develop, and test, methods that explore statistical genetic models reaching beyond additivity when aiming for a more complete dissection of the genetic architecture of complex traits.

The genetic architecture of variation in mean leaf molybdenum concentrations has earlier been explored using GWA analyses in a smaller set of 93 wild collected *A*. *thaliana* accessions [2]. No genome-wide significant associations were found for leaf molybdenum, which was surprising given that the trait has a high heritability [36,43] and that several polymorphisms in *MOT1* are known to contribute to natural variation in this trait [36,37]. When we re-analyzed this data using a method to detect variance differences between genotypes, a strong genetic variance-heterogeneity was identified near the *MOT1* gene [22]. Here, we studied a larger set of 340 *A*. *thaliana* accessions to replicate and fine-map the molecular determinant of this genetic variance-heterogeneity, and find that the strongest associations are to an extended region surrounding *MOT1* (vBLOCK). This is the first successful fine-mapping and replication of a variance-heterogeneity locus on a genome-wide significance scale and in an independent dataset.

In this larger dataset we also identified four loci that independently alter the mean concentration of leaf molybdenum. The minor allele at one of these (*DEL* ^*53*^) was a deletion in the promoter region of *MOT1* previously identified using an F_2_ bi-parental mapping population. This deletion allele decreases the concentration of molybdenum in leaves by down-regulating *MOT1* transcription [36]. Further, we also identified three previously unknown loci, and the minor alleles at these loci (*DUP* ^*326*^, *SNP*_*1*_^*+*^ and *SNP*_*2*_^*+*^) increased the concentration of molybdenum in leaves. One allele (*DUP* ^*326*^) was an insertion polymorphism in the promoter region of *MOT1*, and our analyses revealed that accessions carrying this polymorphism have higher expression of *MOT1* compared to the Col-0 accession that does not carry this polymorphism. The other two associations were to SNPs in regions that were not in LD (r^2^) with the *MOT1* gene or its promoter. One of these SNPs was found ~25 kb downstream of *MOT1* (SNP_1_) and the other ~600 kb upstream of the *MOT1* transcription start-site (SNP_2_). The regulation of molybdenum concentrations in the leaves is hence due to multiple alleles in a gene known to regulate molybdenum uptake, *MOT1*, but also alleles at other neighboring loci that have earlier not been found to contribute to molybdenum homeostasis in *A*. *thaliana*. These results support and refine earlier results from QTL and functional analyses of the *MOT1* region that highlighted the central importance of the *MOT1* region in the regulation of molybdenum homeostasis in natural populations and also suggested that the natural variation in this trait might have a multi-allelic background [36,37]. As it is well known that major loci affecting traits under selection often evolve multiple mutations affecting the phenotype, and that allelic heterogeneity is an important driver of evolution in natural *A*. *thaliana* populations [48], our finding of multiple polymorphisms in this key locus is not surprising. Striking examples of allelic heterogeneity in natural *A*. *thaliana* populations include the large number of different loss-of-function mutants in the *GA5* locus leading to semidwarfs [49], the *MUM2* locus leading to altered seed flotation [50] and the *FRIGIDA* locus leading to an altered flowering-time [51].

Multi-allelic loci are, however, a major challenge in traditional GWA analyses [48]. It is therefore valuable to note that such loci, under certain conditions, can lead to a genetic variance-heterogeneity (see e.g. [10]) that can be detected with a vGWA analysis. The following two examples illustrate how genetic variance-heterogeneity can arise under i) classic allelic heterogeneity where multiple loss-of-function alleles have evolved independently at a locus, and ii) general multi-allelic architectures where the alleles affect the phenotype to various degree and hence either increase or decrease the phenotype relative to that of the major allele. To illustrate how a genetic variance-heterogeneity can emerge under these scenarios, let us consider an example when looking for associations to a bi-allelic SNP with alleles *SNP* ^*A*^ and *SNP* ^*B*^ and where the major SNP allele (*SNP* ^*A*^) is completely linked to the major allele at the functional gene *M* (*M* ^*WT*^). Below, we illustrate how the distribution of the minor alleles across the SNP genotypes will alter the differences in phenotypic mean and variances between the genotypes, and hence affect the power to detect them in GWA and vGWA analyses.

1. If gene *M* evolved via classic allelic heterogeneity, multiple loss-of-function alleles (*M*_*1*_^-^.*M*_*n*_^*-*^) will exist in the population. The largest mean, and smallest variance, difference between the genotype-classes will occur when all n mutant alleles are linked to the *SNP* ^*B*^ allele. As the proportion of the *n M* ^*-*^ alleles linked to the *SNP* ^*A*^ allele increases, the mean difference between genotypes will decrease while the variance differences increase until it reaches its maximum when only one of the *M* ^*-*^ alleles is linked with the *SNP* ^*B*^ allele. In all these scenarios, however, there will be a difference both in the mean and variance between the SNP genotype classes and depending on the power of the study, the locus can be detected by either GWA, or vGWA analyses.
2. If locus M evolved multiple alleles with distinct effects on the phenotype, the locus might display everything from a complete lack of either mean- and variance-effects (scenario (a) below), to both mean and variance effects (b) or variance effects only (c). Under the simplest scenario with two minor alleles, *M* ^*-*^ and *M* ^*+*^, that decreases/increases the trait value relative to that of *M* ^*WT*^, respectively, it is the linkage between the alleles at M and the tested marker that determines the mean and variance differences between the genotypes observed at this locus as shown in the examples below.
  - If the *M* ^*-*^ and *M* ^*+*^ alleles are evenly distributed across the two SNP genotypes, there will neither be a mean nor a variance difference between the genotypes.
  - If the SNP tags the *M* ^*+*^ and *M* ^*-*^ alleles perfectly, i.e. that *SNP* ^*A*^ tags *M* ^*+*^ and *SNP* ^*B*^ *M* ^*-*^ or vice versa, there will be both mean and variance differences between the genotypes.
  - If the *SNP* ^*B*^ allele tags both minor alleles perfectly, i.e. *M* ^*+*^ and *M* ^*-*^ only occurs with *SNP* ^*B*^, there will only be a difference in variance between the SNP genotype classes (S3 Fig).

Hence, the vGWA analysis is likely to be useful for identifying loci under a set of different scenarios ranging from classic allelic heterogeneity to loci with multiple alleles having distinct effects on the phenotype. As shown here, the genetic variance-heterogeneity for vBLOCK was detected based on its genetic variance-heterogeneity due to its close resemblance to scenario (c) above (Fig 2A).

Here, we dissected a locus displaying a genetic variance-heterogeneity for the molybdenum concentration in *A*. *thaliana* leaves into an underlying multi-locus, multi-allelic genetic architecture. We find several alleles at *MOT1* that contribute to this association, which is consistent with findings in earlier studies reporting that several functional variants of this gene alter the mean molybdenum concentrations in *A*. *thaliana* [36,37]. Such multi-allelic architectures, where the different genetic variants affect traits under selection to varying degrees, are not unique to this study but have been described also for other traits and species. For example, in *A*. *thaliana* the *Flowering Locus C* (*FLC*) locus has a natural series of alleles with different effects on vernalization that have been identified [52]. Similar examples also exist in, for example, domestic animal populations for both Mendelian traits, such as coat color [53–55], and complex traits, such as muscularity [56] and meat quality [57]. As illustrated above, the vGWA analysis is a straight-forward and computationally tractable analytical strategy that could be used to identify loci where multi-allelic genetic architectures reduce the additive genetic variance that can be detected by traditional GWA approaches. The examples above suggest that such genetic architectures are likely to be more common than what has been empirically shown to date. We therefore recommend that the vGWA approach be tested on more datasets to reveal how common this type of architecture might be for complex traits. This will also help reveal how large a contribution such multi-allelic genetic architectures contribute to the “missing heritability”.

Little is currently known about the genetic mechanisms contributing to variance-heterogeneity between genotypes in natural populations. Ayroles *et al*. [23] recently reported the first dissection of a locus displaying a genetic variance-heterogeneity in a segregating population and found that mutating a single gene (*Ten-a*) led to a genetic variance-heterogeneity for a behavioral phenotype in *Dropsophila melanogaster*. A number of other, not mutually exclusive, hypotheses have been proposed to explain the origin of genetic variance-heterogeneity at a locus. These can broadly speaking be divided into two categories: those due to the individual locus itself such as multiple functional alleles, incomplete linkage disequilibrium and developmental instabilities [7,10,22], and those due to interactions between the locus and other genetic or environmental factors (i.e. epistasis or gene-by-environment interactions) [8,10,21]. Here, we present the first empirical evidence illustrating how population-wide genetic variance-heterogeneity in a natural population can result from a complex locus involving multiple loci and multiple alleles. We show that this genetic variance-heterogeneity originates from the LD (D’) between multiple functional polymorphisms and the SNP markers defining an LD block around *MOT1* (vBLOCK). The high-variance associated version of this LD-block (*vBLOCK* ^*hv*^) contains three independent polymorphisms (*DEL* ^*53*^, *DUP* ^*326*^ and *SNP*_*1*_^*+*^) altering the molybdenum concentration in leaves relative to the major alleles at these loci on the low-variance associated version (*vBLOCK* ^*lv*^). Two of these polymorphisms increase molybdenum and one decrease it, leading to a highly significant genetically determined variance-heterogeneity amongst the accessions that share *vBLOCK* ^*hv*^ (Fig 2A; multi-allelic example c above). Our work also illustrates how the use of alternative genetic models in GWA analyses can provide novel insights to complex genetic architectures underlying adaptively important traits in natural populations.

The LD (D’) between multiple functional polymorphisms and vBLOCK in this collection of natural *A*. *thaliana* accessions is the key genomic feature that facilitated the discovery of this locus in the vGWA. Although the molecular basis for this LD-pattern, as well as the reasons for multiple independent polymorphisms being found almost exclusively with one of the variants of this LD-block, is unknown, it is interesting to note that they could have emerged via the processes discussed in relation with the appearance of synthetic LD in GWA studies [58]. It would therefore be interesting to, in the future, explore whether the same basic genomic processes might drive the emergence of both synthetic and vGWA associations in general, or whether the resemblance between the genetic architecture described here and the mechanism proposed by Dickson *et al*. [58] is a rare case of where the two overlap.

Many GWA studies have found that the total additive genetic variance of associated loci is considerably less than that predicted based on estimates of the narrow-sense heritability, i.e. the ratio between the additive genetic and phenotypic variance in the population. This common discrepancy between the two is often called the curse of the “missing heritability” and is viewed as a major problem in past and current GWA studies [59]. Here, we provide an empirical example of how a vGWA is able to identify a locus [22] that remained undetected in a standard GWA [2] and that, when the underlying genetic architecture was revealed, was found to make a large contribution to the additive genetic variance and narrow-sense heritability. This illustrates the importance of utilizing multiple statistical modeling approaches in GWA studies to detect the loci contributing to the phenotypic variability of the trait, and then also continue to further dissect the underlying genetic architecture to uncover how the loci potentially contribute to the heritability that was “missing” in the original study [2].

By evaluating T-DNA insertional alleles of genes in LD with the SNPs associated to leaf molybdenum concentrations, we are able to suggest two novel functional candidate genes involved in molybdenum homeostasis in *A*. *thaliana*. Little is known about the function of one of these, *AT2G27030*, and further work is needed to explore the mechanisms by which it may alter molybdenum concentrations in the plant. The second gene (*AT2G26975*; Copper Transporter 6; *COPT6*) located ~600 kb upstream of *MOT1* is from earlier studies known to be involved in the connected regulation of copper and molybdenum homeostasis in plants. It was recently reported [46] that *MOT1* and several copper transporters were up-regulated under copper deficiency in *B*. *napus*, suggesting a common regulatory mechanism for these groups of genes. Further experimental work is needed to explore the potential contributions of these genes to natural variation in molybdenum homeostasis, and the potential connection between copper and molybdenum homeostasis.

Here, we dissect a complex locus affecting molybdenum concentration in the *A*. *thaliana* leaf and find it likely that three closely linked genes contribute to this effect. Clustering of genes with similar function is well known for Resistance (R) genes [60] and close linkage between genes important for growth rate has also been evidenced [61] in *A*. *thaliana*. How common such functional clustering into complex loci will be for traits of importance for evolution is still largely unknown as the resolution in most complex trait studies does not allow the separation of effects from closely linked loci. Our finding that not only the already known gene in this region, *MOT1*, but likely also other novel genes contribute to the diverse range of molybdenum concentrations in the leaf observed in this collection of natural *A*. *thaliana* accessions suggest that the clustering of loci has been of adaptive value for this ecologically relevant trait. This makes the locus a highly interesting candidate for future work to better understand the role of gene clustering for the evolution of adapted populations.

In summary, here we dissect a locus displaying a genetic variance-heterogeneity for leaf molybdenum concentration in *A*. *thaliana* [22] into the contributions from three independent alleles that are in high LD with the high-variance associated version of an extended LD-block surrounding the *MOT1* gene. This is the first empirical example of how a multi-locus, multi-allelic genetic architecture can lead to genetic variance heterogeneity at a locus. The dissection of the genetic architecture underlying the vGWA signal allowed the transformation of non-additive genetic variance into additive genetic variance, and hence allowed the detection of a significant part of the “missing heritability” in the variation in leaf molybdenum concentrations in this species-wide collection of *A*. *thaliana* accessions. This study also delivers insights into how vGWA mapping facilitates the detection and genetic dissection of the genetic architecture of loci contributing to complex traits in natural populations. It thereby illustrates the value of using alternative statistical methods in genome-wide analyses. Further, it provides an approach to infer multi-allelic loci, which are likely to be both a common, and far too often ignored, complexity in the genetics of multifactorial traits that contributes to undiscovered additive genetic variance and consequently the curse of the “missing heritability”.

## Materials and Methods

### Genotype and phenotype data

The concentration of molybdenum in leaves was measured in 340 natural *A*. *thaliana* accessions from the ‘HapMap’ collection ([3]; S1 Table). This dataset contains 58 of the 93 accessions used in the earlier GWA [2] and vGWA [22] analyses of leaf molybdenum concentrations supplemented with 282 newly phenotyped accessions. All accessions were grown in a controlled environment with 6 biological replicate plants per accession, and analyzed by Inductively Coupled Mass Spectroscopy (ICP-MS) for multiple elements including molybdenum, as described previously by Baxter *et al*. [3]. All the ICP-MS data used for the GWA and vGWA is accessible using the digital object identifier (DOI) 10.4231/T9H41PBV, and data for the evaluation of candidate genes using T-DNA insertional alleles is accessible using the DOI 10.4231/T9NP22C0 (see http://dx.doi.org/).

All accessions have previously been genotyped using the 250k *A*. *thaliana* SNP chip and that data is publicly available [3]. SNPs where the minor allele frequency was below 5% were excluded from the analyses. Genotypes were available for more than 95% of the SNPs in all accessions, so none were removed due to problematic genotyping. In total, 200,345 SNPs passed this quality control and were used in our GWA and vGWA analyses.

We evaluated the region upstream of *MOT1* for structural polymorphisms in a set of 283 accessions selected to cover the range of leaf molybdenum concentrations (S5 Table). This was done using gel electrophoresis to identify PCR fragment size differentiation using the primers described in S6 Table. The PCR reactions were completed as follows: 1μl DNA + 5X GoTaq Bf, 2.5mM dNTP’s, 25mM MgCl_2_, 0.4μM of each primer, 0.3μl Taq polymerase, and 9.7μl nuclease free water for a total reaction volume of 25μl. PCR conditions were 94°C for 1 minute to denature, 54°C for 1 minute to anneal, and 72°C for 1.25 minutes for extension, repeated for 40 cycles in the Thermo Px2 thermal cycler (Electron Corporation). DNA was prepared for the accessions that displayed suggestive evidence for structural polymorphisms and submitted for sequencing using Macrogen (dna.macrogen.com). The sequences were then compared to the Col-0 reference sequence using DiALIGN (http://bibiserv.techfak.uni-bielefeld.de/dialign/), which uncovered five loci and six segregating structural polymorphisms (S2 Table) that were then genotyped in the 283 phenotyped accessions (S5 Table).

### Statistical analyses

All analyses described in the sections below were performed using the R-framework for statistical computing [62]. All figures, except Fig 2, were prepared using R.

#### GWA and vGWA analyses

The variance-heterogeneity genome-wide association analyses (vGWA) were performed using Squared residual Value Linear Modeling, SVLM, as implemented in the VariABEL R-package [63]. In short, this two is a two-step method where the trait is first adjusted for a potential mean SNP effect and other covariates in a regression analysis, and then a second regression analysis is applied to the squared residual values from the first analysis, using the SNP as the predictor. This analysis will identify any potential genetic variance-heterogeneity at a locus as the variance for each genotype is equal to the mean of the squared residual of the trait conditional on genotype. To control for population-structure, Grammar+ residuals were used as phenotypes in these analyses [64]. The Grammar+ residuals were calculated using a linear mixed model, incorporating the IBS-matrix to correct for population stratification, using the *polygenic* function implemented in the R-package GenABEL [65].

The genome-wide association (GWA) analyses were performed using a linear mixed model, incorporating the IBS-matrix to correct for population stratification, via the *polygenic* and *mmscore* functions implemented in the R-package GenABEL [65].

A genome-wide significance threshold was determined for all tested phenotypes by Bonferroni-correction for the number of tested SNPs, resulting in a threshold of 2.5 × 10^−7^. To detect potential inflation of the p-values in the GWA analyses due to remaining population stratification and/or cryptic relatedness, we visually evaluated the relationship between the theoretical distribution of p-values under the null-hypothesis versus those observed in the GWA using quantile-quantile (QQ) plots (S4 Fig), and calculated the inflation factor using the function *estlambda* in the GenABEL package [65].

#### Multi-locus LASSO regression analyses

Multi-locus regression analysis to identify independent SNP effects on leaf molybdenum concentrations was performed using LASSO regression implemented in the R-package *glmnet* [45]. To control for population-structure, Grammar+ residuals were used as phenotypes in these analyses [64]. The LASSO analysis identifies the linear model that minimizes the following $\sum_{i}{\left(y_{i}-{\hat{y}}_{i}\right)}^{2}+\lambda \sum_{j}\left(\beta_{j}\right)$ where *y*_*i*_ and ${\hat{y}}_{i}$ is the phenotype and the predicted phenotype of individual *i*. *β*_*j*_ is the individual genotype effects. The constraint will force most genotype effects to zero, thereby identifying a small subset of polymorphisms with strong independent effects on the phenotype. As *λ* decreases, the number of non-zero estimates will increase. If *λ* is zero, the method is identical to an ordinary linear regression. Here, we empirically selected a *λ* where all SNPs with non-zero effects reached the genome-wide significance threshold in the GWA or vGWA analysis (S1 Fig).

#### DGLM analyses to simultaneously estimate mean and variance effects of evidenced loci

Within the Double Generalized Linear Model (DGLM) framework it is possible to simultaneously model both dispersion and mean by fitting separate linear predictors for them [66,67]. We fitted a DGLM with separate genetic effects for the variance, and for the mean:

$Y∼N(X_{1}\beta_{1},e^{X_{2}\beta_{2}})$ where *Y* is the Grammar+ residuals for the molybdenum concentrations used to control for population-structure in the analyses [64], *X*_1_ = [*SNP*_1_, *DEL*, *DUP*]^*T*^, *X*_2_ = *SNP*_*i*_ and *i* is the index of the SNP whose variance effect we are estimating. *β*_1_ and *β*_2_ were estimated using maximum likelihood. The model was fitted using the R-package *dglm* [67] as suggested in [10]. It should be noted that although the DGLM analysis is very useful for disentangling mean and variance effects of loci, it is not optimal for genome-wide analyses as it is both computationally demanding and provides highly conservative genome wide p-values (*λ* = 0.75 for leaf molybdenum concentration in this population). Here, DGLM was used to i) re-scan the vGWA region on chromosome 2 to identify the SNP with the strongest variance effect in vBLOCK and ii) include evidenced loci as co-factors with mean effect, while redoing the vGWA scan to evaluate whether the loci identified in the GWA led to the vGWA association.

#### Heritability estimates

Every accession in our data was grown with at least 6 replicates plants. The broad sense heritability (H^2^) was calculated using an ANOVA *y* = *β*_0_ + *accession* × *β*_*acc*_ + *e*, comparing within and between line variances.

To calculate the narrow sense heritability (h^2^) we fitted a mixed model $\bar{y}=\mu +Zb+e$. Here $\bar{y}$ is the mean leaf molybdenum concentration per line and *ZZ* ^*T*^ = *G*, where *G* is the genomic kinship matrix. The intra-class correlation $r=\frac{\sigma_{b}^{2}}{\sigma_{b}^{2}+\sigma_{e}^{2}}$ given by this model tells us the amount of variance in $\bar{y}$ explained by kinship. Assuming that the within line replicates has removed all environmental variance, the amount of the total phenotypic variance explained by kinship, aka h^2^, is *rH* ^2^. In reality, as $\bar{y}$ is estimated using <10 replicates for most lines, some environmental noise will remain in $\bar{y}$, in which case *rH* ^2^ ≤ *h* ^2^ ≤ *r*. Here, we therefore present the *rH* ^2^ values, which is the lower bound of h^2^.

#### Variance explained

We estimated the fraction of H^2^ explained by the markers in the *MOT1* region as $R^{2}=1-\frac{var(\bar{y}-X\beta )}{var(\bar{y})}$, where $\bar{y}$ is the mean molybdenum content per line and *X* is the genotype matrix for the markers, fitted as a fixed effect. This estimate assumes that $\bar{y}$ contains no environmental variance which, as stated above, is not entirely the case. If $\bar{y}$ contains environmental noise, this estimate will instead be the lower bound of the fraction of H^2^ explained by *X*, in the same way as described above for h^2^.

The fraction of h^2^ explained by the evaluated set of polymorphisms in the *MOT1* region was estimated by comparing two mixed models:

$$\bar{y}=\mu +Zb+e$$ (1)

$$\bar{y}=X\beta +Zb+e$$ (2)

The intra-class correlation *r*_1_ in model (1), gives the amount of variance in $\bar{y}$ explained by kinship, whereas the intra-class correlation *r*_2_ in model (2) gives the amount of residual variance explained by kinship in this model. To compare the two, we calculate the amount of variance in $\bar{y}$ explained by kinship under model (2) as *r*_2,*tot*_ = *r*_2_(1 − *R* ^2^). The fraction of h^2^ explained by the fixed effects *X* are then given as $\frac{r_{1}-r_{2,tot}}{r_{1}}$. The fraction of variance explained by *X* that is additive is calculated as $\frac{r_{1}-r_{2,tot}}{R^{2}}$.

#### Expression analysis to evaluate the potential effects of the associated *MOT1* promoter polymorphisms

We quantified the levels of *MOT*1 mRNA in roots of 6 accessions carrying the *DUP* ^*326*^ polymorhism, 5 accessions carrying the *DEL* ^*53*^ polymorphism and Col-0 as a reference (S3 Table) using a protocol similar to that of [38]. Roots from plants grown under identical conditions to those used for ICP-MS analysis were separated from the shoots and rinsed thoroughly with deionized water to remove any soil contamination. The samples were frozen in liquid nitrogen and stored at -80°C until extraction. Total RNA was extracted, and DNase digestion was performed during the extraction, using the Invitrogen PureLink RNA Mini Kit. Two micrograms of total RNA were used a template to synthesize first-strand cDNA with random hexamers, using SuperScript II Reverse Transcriptase (Invitrogen Life Technologies). Quantitative real-time PCR (qRTPCR) was performed with first strand cDNA as a template on three independent biological samples for each accession, using a sequence detector system (StepOne Plus, Applied Biosystems). For normalization across samples within a qRT-PCR run, the expression of either *PP2A* or *UBQ10* was used. For quantification of *MOT1* the following primers were used: forward primer 5’-GGT GGG TGT GTG GCA CTG T-3’ and reverse primer 5’-AGC ACA CCA ACC GGA AAC TT-3’. The cycle threshold (C^T^) values were determined based on efficiency of amplification. The C^T^ values were normalized against the mean expression of either *PP2A* or *UBQ10* by calculating ΔC^T^ values as C^T^_MOT1_–mean(C^T^_PP2A(UBQ10)_). The relative change in *MOT1* expression versus Col-0 was then calculated for every accession i as ΔΔC^T^_i_ = ΔC^T^_i_—ΔC^T^_Col-0_. The fold change in expression for accession *i* was then calculated as 2^−ΔΔCT,i^.

#### Functional evaluation of candidate genes using T-DNA insertion lines

We identified all genes in the LD-region (r^2^ > 0.4) surrounding the SNP_1_ and SNP_2_ loci. T-DNA insertional alleles, catalogued as homozygous at T-DNA Express (http://signal.salk.edu/cgi-bin/tdnaexpress), were ordered for all genes where they were available (Table 3; S4 Table) from the Nottingham Arabidopsis Stock Centre (NASC) with the exception of the GABI-kat lines which were received from the stock centre as F3 families. Since *MOT1* is known to regulate molybdenum concentrations in *A*. *thaliana*, the *mot1*-1 T-DNA insertional allele (SALK_118311) for this gene was included on every experimental block of plants as a control, along with wild-type Col-0. An experimental block is defined by a cultivation tray containing 9 genotypes (including wild-type Col-0 and *mot1-1*) with each genotype represented by between 2–12 individuals per block. The tested T-DNA insertional alleles were grown in 8 independent blocks and the molybdenum concentration in leaves of all plants quantified by ICP-MS using the same procedure as used previously [36].

For every experimental block, we compared the molybdenum concentration in leaves between the replicates of every T-DNA insertion line, versus wild type Col-0, using the non-parametric Wilcox rank test. In 6 out of the 8 blocks, *mot1-1* showed significantly lower molybdenum concentrations compared to the wild type Col-0 (p < 0.05) as expected, and in one block, the reduction was significant at (p < 0.1). The *mot1-1* mutant in one experimental block of plants showed no difference compared to the wild type Col-0, and the results for the genotypes in this experimental block that were not supported by the results in other experimental blocks were discarded. To combine the data on T-DNA alleles that were replicated on several experimental blocks, we normalized molybdenum concentrations against wild type Col-0 within blocks and jointly analyzed the normalized values using the Wilcox rank test.

### Explorations of the long-range LD-block surrounding the *MOT1* gene

The vGWA analyses identify a strong variance-heterogeneity signal across a number of markers on chromosome 2 that contains the functional candidate *MOT1* gene. The LD is high among these significant markers that define an extended vGWA associated vBLOCK. Visual inspection of the genotype-matrix of this region, sorted by the genotype of the leading SNP in the vGWA analysis (Table 1), indicated the presence of two major groups of accessions that carry the same alleles across a large number of the associated markers (Fig 1C).

## Supporting Information

S1 Fig Selection of penalty (λ) in LASSO analysis. Penalty is selected such that all SNPs with non-zero effects in the analysis have reached the genome-wide significance threshold in the GWA or vGWA analysis.
(TIFF)

S2 Fig Evaluated T-DNA mutants in region near SNP_1_. We identified the genes (colored boxes) in the regions surrounding SNP1 that were bounded by the furthest up- and downstream SNPs with r^2^ > 0.4. We measured the mean leaf molybdenum concentrations for available T-DNA insertion lines and compared them to the reference genotype (Col-0). Yellow box = nominally significant difference in leaf molybdenum concentration, deep blue box = no significant difference, light blue = no T-DNA insertion line tested.
(TIFF)

S3 Fig An illustration of how a multi-allelic genetic architecture could lead to variance- heterogeneity signals in a population. The top panel illustrates the hypothetical phenotypic distributions three alleles–*M* ^*-*^, *M* ^*WT*^ and *M* ^*+*^—that have different effects on a hypothetical trait. The bottom panel illustrate the mixture distributions observed in an association analysis to a bi-allelic marker, where one of the marker- alleles tag functional allele *M* ^*WT*^, and the other tag both alleles *M* ^*-*^ and *M* ^*+*^. In this situation, no mean difference could be observed between the marker alleles, whereas a large variance difference could be detected via the variance-heterogeneity between the SNP genotypes using a vGWA analysis.
(TIFF)

S4 Fig Quantile-quantile (QQ) plots for the genome-wide association analyses to detect genetic effects on the trait mean (GWA), or variance (vGWA). Black line illustrates the theoretical distribution of p-values under the null-hypothesis and the blue dots those observed in the two analyses.
(TIFF)

S1 Table Accessions phenotyped for molybdenum content. (XLSX)

S2 Table Structural polymorphisms in MOT1 promoter region. (XLSX)

S3 Table Summary statistics and qRT-PCR data to estimate expression of MOT1 in accessions carrying either *DUP* ^*326*^ or *DEL* ^*53*^
(XLSX)

S4 Table Tested T-DNA insertion lines. (XLSX)

S5 Table MOT1 promoter polymorphism genotypes for 283 A. thaliana accessions. (XLSX)

S6 Table Primers for genotyping the promoter region of MOT1. (XLSX)

S1 Text Testing for within versus between line variance heterogeneity. (DOCX)
