# Supplementary figures and images for: The Multi-allelic Genetic Architecture of a Variance-Heterogeneity Locus for Molybdenum Concentration in Leaves Acts as a Source of Unexplained Additive Genetic Variance
Source: PLoS Genet. 2015 Nov 23;11(11):e1005648. doi: 10.1371/journal.pgen.1005648 (PMC4657900; doi:10.1371/journal.pgen.1005648)

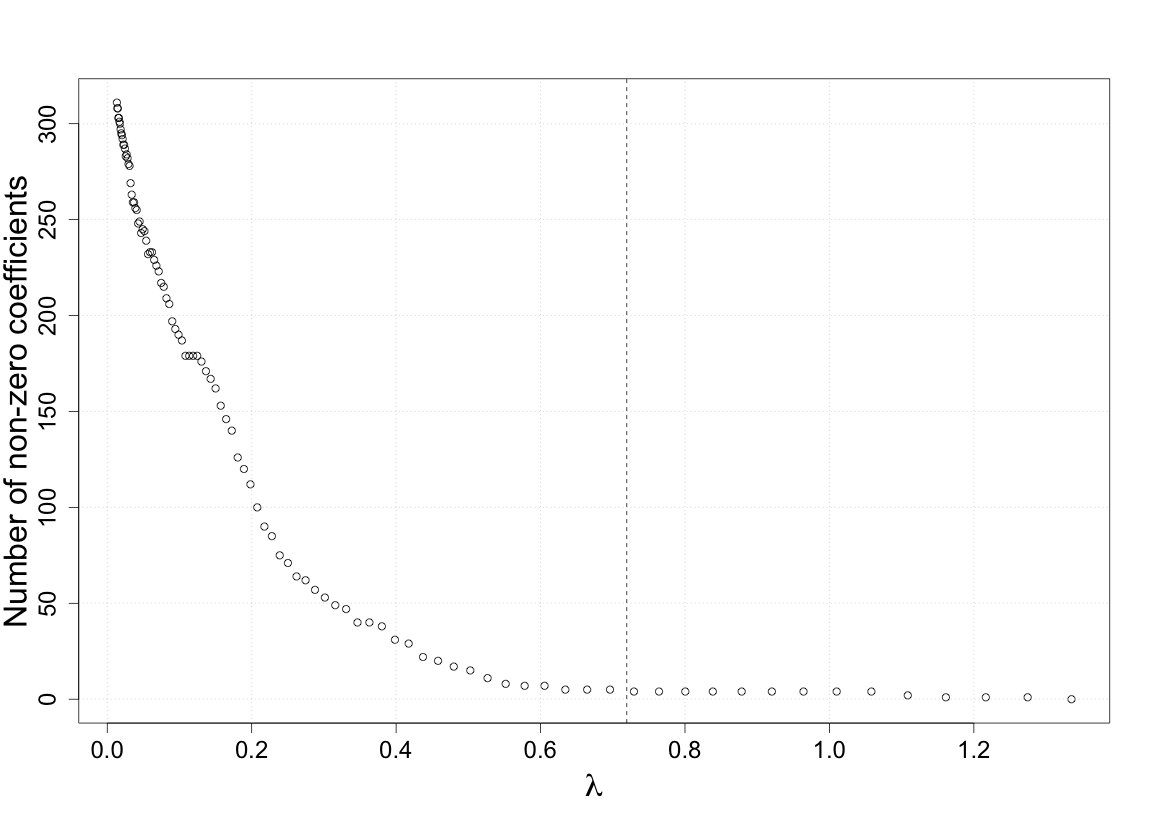

Supplement: S1 Fig — Penalty is selected such that all SNPs with non-zero effects in the analysis have reached the genome-wide significance threshold in the GWA or vGWA analysis. (TIFF) [file pgen.1005648.s001.tiff]

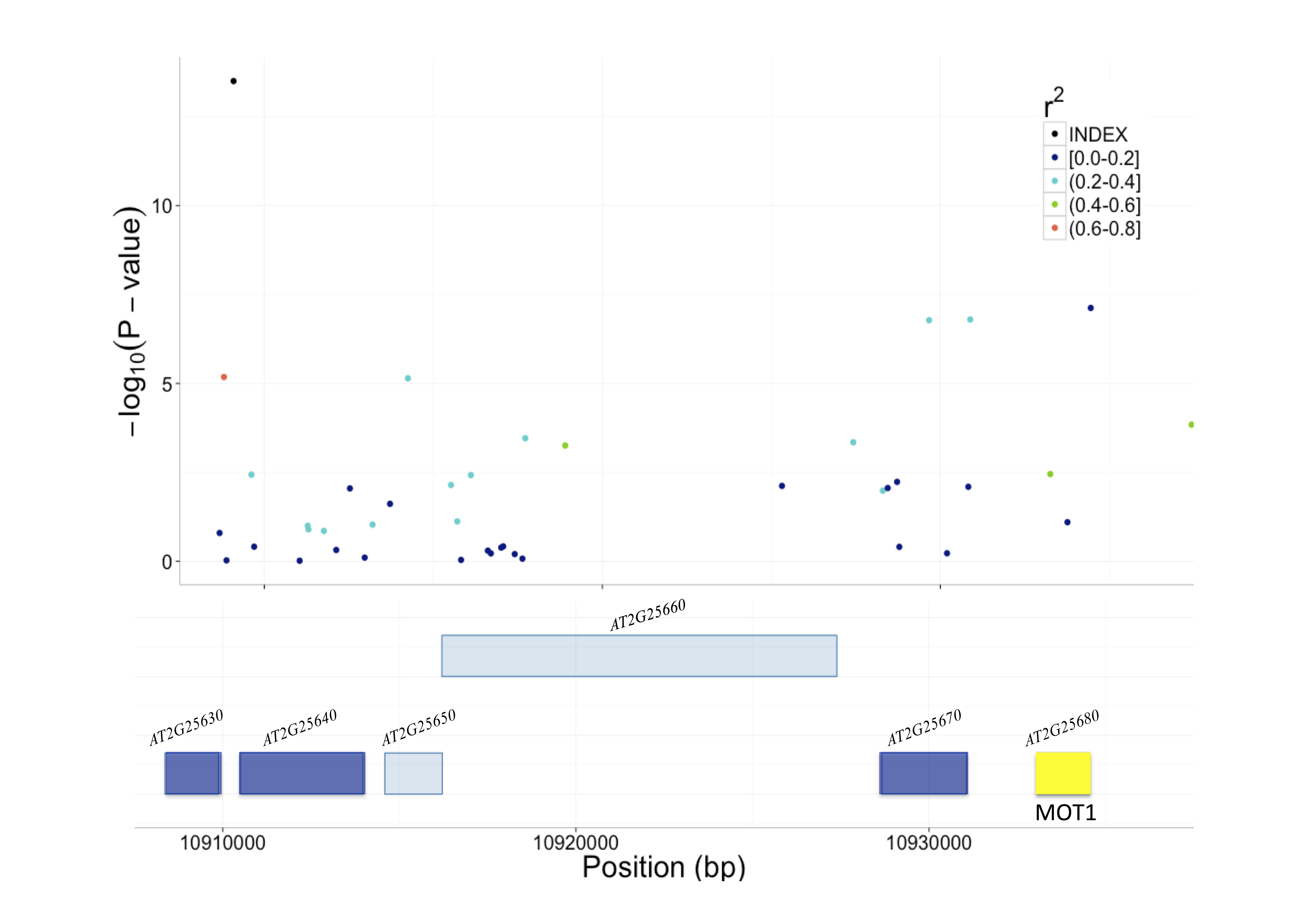

Supplement: S2 Fig — We identified the genes (colored boxes) in the regions surrounding SNP1 that were bounded by the furthest up- and downstream SNPs with r2 > 0.4. We measured the mean leaf molybdenum concentrations for available T-DNA insertion lines and compared them to the reference genotype (Col-0). Yellow box = nominally significant difference in leaf molybdenum concentration, deep blue box = no significant difference, light blue = no T-DNA insertion line tested. (TIFF) [file pgen.1005648.s002.tiff]

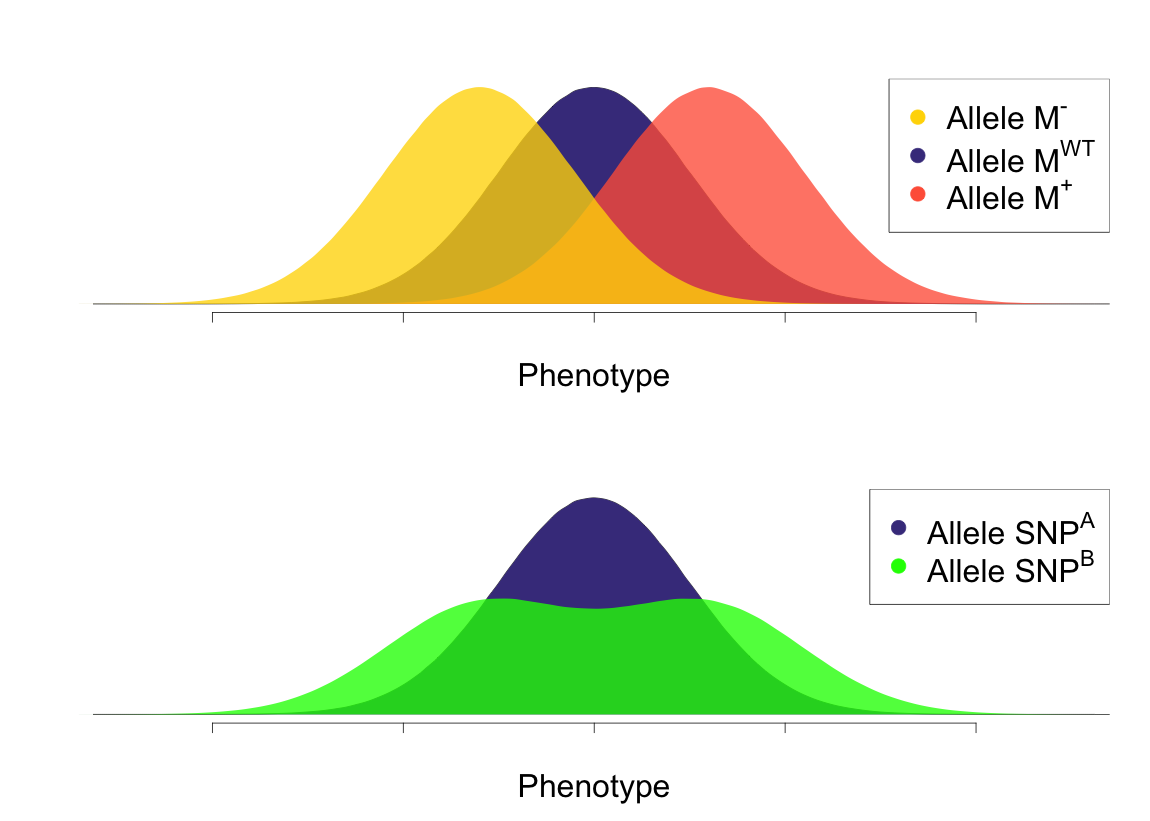

Supplement: S3 Fig — The top panel illustrates the hypothetical phenotypic distributions three alleles–M -, M WT and M +—that have different effects on a hypothetical trait. The bottom panel illustrate the mixture distributions observed in an association analysis to a bi-allelic marker, where one of the marker- alleles tag functional allele M WT, and the other tag both alleles M - and M +. In this situation, no mean difference could be observed between the marker alleles, whereas a large variance difference could be detected via the variance-heterogeneity between the SNP genotypes using a vGWA analysis. (TIFF) [file pgen.1005648.s003.tiff]

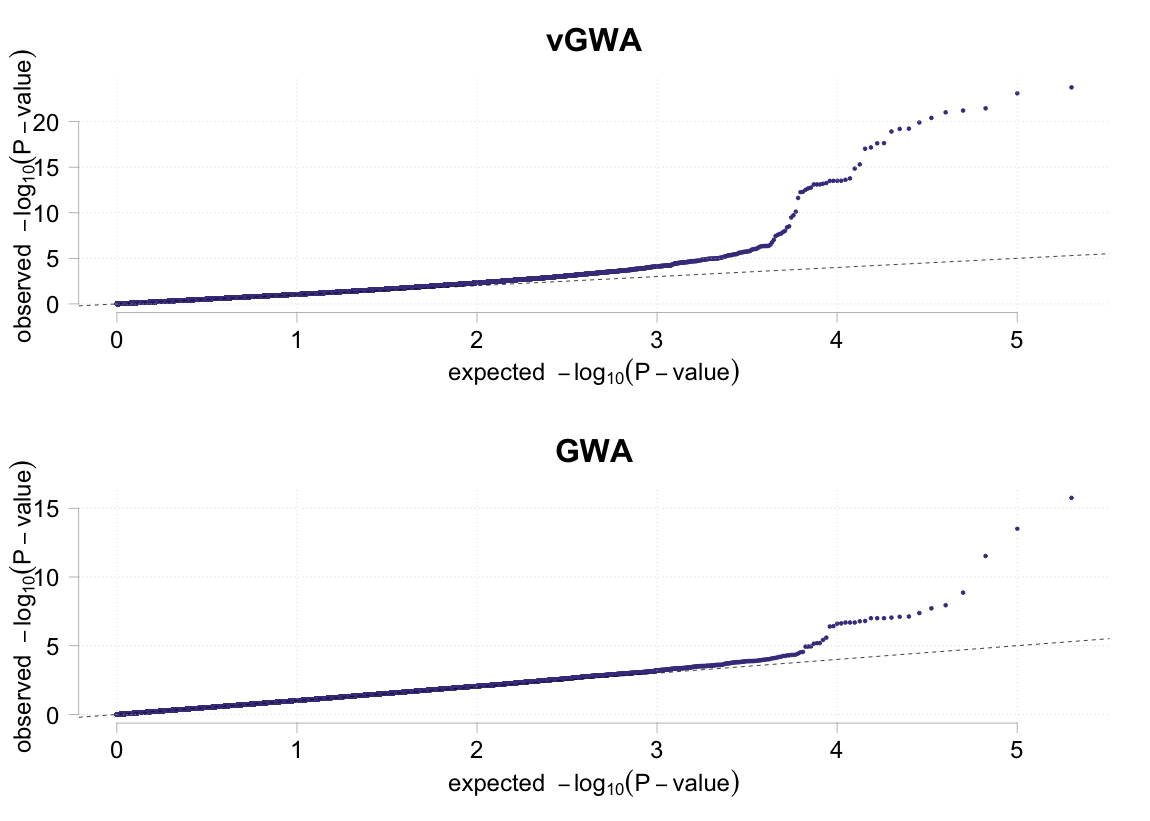

Supplement: S4 Fig — Black line illustrates the theoretical distribution of p-values under the null-hypothesis and the blue dots those observed in the two analyses. (TIFF) [file pgen.1005648.s004.tiff]
