# Supplementary material for: The Multi-allelic Genetic Architecture of a Variance-Heterogeneity Locus for Molybdenum Concentration in Leaves Acts as a Source of Unexplained Additive Genetic Variance
Source: PLoS Genet. 2015 Nov 23;11(11):e1005648. doi: 10.1371/journal.pgen.1005648 (PMC4657900; doi:10.1371/journal.pgen.1005648)
Supplement: S1 Text — (DOCX) [file pgen.1005648.s011.docx]

**S1 Text: Testing for within versus between line variance heterogeneity**

Depending on the underlying genetic architecture, vQTLs can manifest themselves as either within- and/or between-line genetic variance heterogeneity. In the main text, we show that the vQTL at the *MOT1* locus is caused by a multi-allelic genetic architecture. This is an example where the allelic heterogeneity at this locus across the lines in the analysed populations leads to a population-wide between-line genetic variance heterogeneity.

An alternative genetic mechanism that can lead to a genetic variance heterogeneity at a locus is the scenario where individuals carrying one allele at the vQTL respond differently to, for example, stochastic fluctuations in the environment than the individuals carrying the other allele. This scenario is sometimes refered to as allelic plasticity. When the genetic variance-heterogenetiy is due to this type of mechanism, it will generally lead to there being both a within- and between-line genetic variance heterogeneity in the population. It can therefore be separated from the mechanisms leading to between line genetic variance heterogeneity only, such as multi-allelic genetic architectures similar to that reported here for *MOT1*, as it is the only one leading to both within- and between line variance-heterogeneity in the vGWA analyses.

When doing a vGWA, one typically searches for between-genotype variance-heterogeneity, as described in the Results and Methods sections in the main text. In order to also detect within-line genetic variance heterogeneity, it is necessary to have replicate measurements per genotype. Such data is often available for inbred model organisms, but rarely in higher, non-inbred organisms. Our dataset is an example where multiple replicates are available for each inbred accession. We could therefore also test for the presence of a within-line genetic variance heterogeneity, quantified via the within-line coefficient of variation (CV) of molybdenum concentrations, across the vQTL in the vBLOCK region. By using the CV for each line as the phenotype in a GWA analysis performed as described in the Methods section of the main text, we could test for the presence of any within-line genetic variance-heterogeneity. Given that we have already shown that the vQTL in this region is due to a multi-allelic genetic architecture at the locus (see Main text), no within-line genetic variance heterogeneity was expected in this region. This is also what was found in the GWA analysis where no significant genetic effects were found for the CV in the vBLOCK region.
